# Effect of LRRK2 protein and activity on stimulated cytokines in human monocytes and macrophages

**DOI:** 10.1038/s41531-022-00297-9

**Published:** 2022-03-28

**Authors:** Diba Ahmadi Rastegar, Laura P. Hughes, Gayathri Perera, Shikara Keshiya, Siying Zhong, Jianqun Gao, Glenda M. Halliday, Birgitt Schüle, Nicolas Dzamko

**Affiliations:** 1 University of Sydney, Brain and Mind Centre and Faculty of Medicine and Health, School of Medical Sciences, Camperdown, NSW 2050 Australia; 2 Department of Pathology, Stanford School of Medicine, Stanford, CA 94305 USA

**Keywords:** Parkinson's disease, Inflammation

## Abstract

Leucine-rich-repeat kinase 2 (LRRK2), a potential therapeutic target for the treatment of Parkinson’s disease (PD), is highly expressed in monocytes and macrophages and may play a role in the regulation of inflammatory pathways. To determine how LRRK2 protein levels and/or its activity modulate inflammatory cytokine/chemokine levels in human immune cells, isogenic human induced pluripotent stem cells (iPSC) with the LRRK2-activating G2019S mutation, wild-type LRRK2, and iPSC deficient in LRRK2 were differentiated to monocytes and macrophages and stimulated with inflammatory toll-like receptor (TLR) agonists in the presence and absence of LRRK2 kinase inhibitors. The effect of LRRK2 inhibitors and the effect of increasing LRRK2 levels with interferon gamma on TLR-stimulated cytokines were also assessed in primary peripheral blood-derived monocytes. Monocytes and macrophages with the LRRK2 G2019S mutation had significantly higher levels of cytokines and chemokines in tissue culture media following stimulation with TLR agonists compared to isogenic controls. Knockout of LRRK2 impaired phagocytosis but did not significantly affect TLR-mediated cytokine levels. Interferon gamma significantly increased the levels of LRRK2 and phosphorylation of its downstream Rab10 substrate, and potentiated TLR-mediated cytokine levels. LRRK2 kinase inhibitors did not have a major effect on TLR-stimulated cytokine levels. Results suggest that the LRRK2 G2019S mutation may potentiate inflammation following activation of TLRs. However, this was not dependent on LRRK2 kinase activity. Indeed, LRRK2 kinase inhibitors had little effect on TLR-mediated inflammation under the conditions employed in this study.

## Introduction

Genetic variation in leucine-rich-repeat kinase 2 (LRRK2) is strongly implicated in the risk of developing the neurodegenerative movement disorder Parkinson’s disease (PD). At least six missense mutations in LRRK2 increase the enzyme’s kinase activity^1–3^, and comprise a frequent cause of autosomal dominantly inherited PD^4^. Common variation in *LRRK2* is also associated with a greater risk of idiopathic PD^5–8^, and increased LRRK2 activity has been measured in brain tissue from PD patients without LRRK2 mutations^9^. As increased LRRK2 activity is pathogenically linked to PD, much effort has gone into the development of small-molecule LRRK2 inhibitors as potential therapeutics^10,11^. Preclinical studies have suggested the potential efficacy of LRRK2 inhibitors across different models, and indeed, the first LRRK2 inhibitors are currently in early phase clinical trials^12–14^. However, the biological function of LRRK2 remains unclear and exactly how LRRK2 contributes to the pathogenesis of PD is unknown.

One intriguing aspect of LRRK2 biology is its much higher expression in peripheral immune cells compared to central nervous system cells. Indeed, accumulating evidence indicates a role for LRRK2 in immune cell function^15,16^. LRRK2 has a particularly high expression in monocytes and neutrophils^17–21^ and is phosphorylated by IkB kinase family members following activation of toll-like receptors (TLRs)^22^. LRRK2 kinase activity has also been implicated in the activation of inflammasome complexes^23^ and the host response to ligands of the dectin-1 receptor^24,25^. Moreover, *LRRK2* variation has also been genetically linked to the susceptibility of infection from *M. leprae*^26^, and a number of studies have now functionally linked LRRK2 to the clearance of pathogens such as *M. tuberculosis*^27,28^ and *S. typhimurium*^17,29^. Finally, LRRK2 variation has also robustly been linked to the risk of developing inflammatory bowel disease^30^. Collective studies therefore clearly implicate LRRK2 in innate immune responses^31^.

A major aspect of the innate immune pathway is to modulate the host response to pathogens via the production of inflammatory cytokines. Studies predominantly using mice or murine derived immune cells have suggested a role for LRRK2 in the regulation of inflammatory cytokine production^32^. This is likely of importance as increasing evidence continues to implicate inflammation in the early pathogenesis of PD^33–37^. Indeed, the assessment of inflammatory cytokine profiles in LRRK2 G2019S mutation carriers found elevated serum levels of IL1β, TNFα, IL6 and IL12 in a subset of asymptomatic mutation carriers compared to controls^38^, and LRRK2 patients with more severe symptoms present with higher levels of the proinflammatory proteins IL8, MCP1 and MIP1β^39^. Moreover, frequent use of non-steroidal anti-inflammatory medication is associated with reduced penetrance of PD in LRRK2 mutation carriers^40^. Despite the evidence supporting a role for LRRK2 function in the immune system, exactly how LRRK2 modulates inflammatory signalling in human cells is still unclear. We have therefore employed isogenic induced pluripotent stem cell lines (iPSC) with the pathogenic G2019S mutation, LRRK2 knockout, and LRRK2 wild type and differentiated them to monocytes and macrophages. We find evidence that the LRRK2 G2019S mutation can potentiate TLR-mediated cytokines, but we find little effect of LRRK2 inhibitors or LRRK2 knockout on the TLR-stimulated cytokine response.

## Results

### Differentiation of LRRK2 iPSCs to monocytes and macrophages

**Fig. 1:**
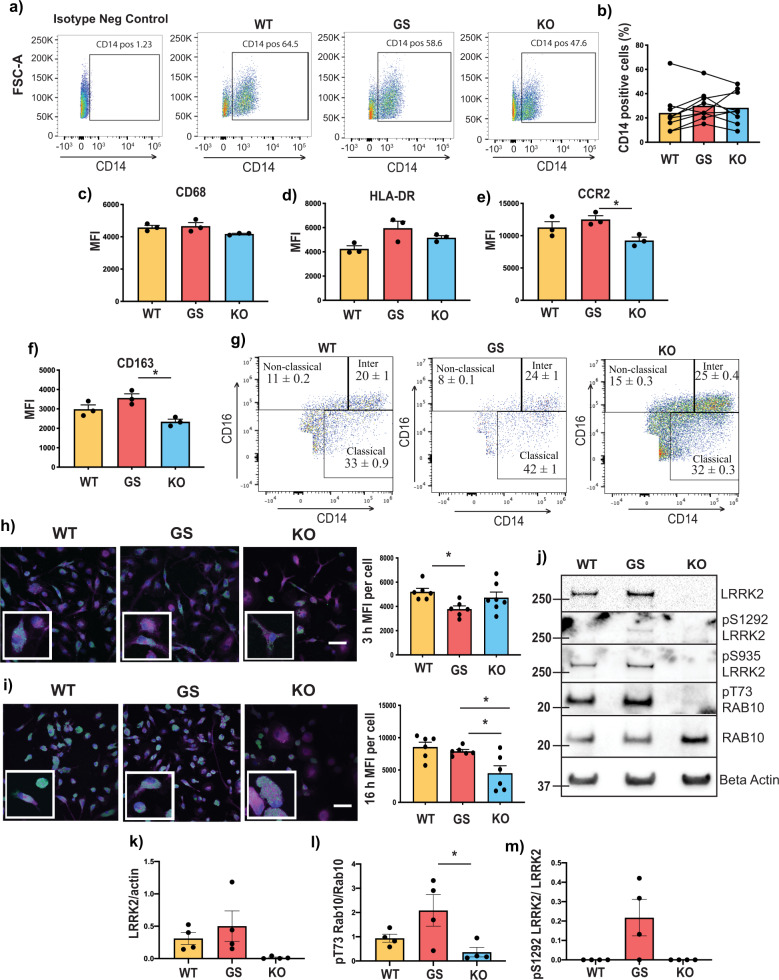
Characterisation of IPS-derived monocytes. Flow cytometry was used to measure the number of cells expressing the monocyte marker CD14 in collected tissue media following monocyte differentiation with representative flow cytometry plots shown in (**a**). Up to 100,000 events were generally captured. Flow cytometry results were then quantified (**b**), with the graph showing the mean percent of CD14 cells in the tissue culture media, while the joined dots indicate specific results for each genotype for the different differentiation rounds. Flow cytometry was also used to measure the expression of monocyte markers CD68 (**c**), HLA-DR (**d**), CCR2 (**e**) and CD163 (**f**) after gating on CD14-positive monocytes. Graphs show mean ± SEM with dots indicating triplicate measurements. Results are representative of at least 2 biological replicates. **g** CD14 and CD16 were used to determine the percent of classical, intermediate and non-classical monocytes in the different LRRK2 genotype cell lines. The uptake of GFP-labelled latex beads, shown in green in the representative micrographs was determined in differentiated macrophages using immunofluorescence at 3 h (**h**), and 16 h (**i**). Cell mask, shown in magenta, was used to visualise internalised GFP signal. The blue staining is DAPI. The scale bar = 20 μm. Graphs indicate the median fluorescence intensity (MFI) of GFP puncta per cell ±SEM. Dots indicate the results from each image analysed, with each image comprising ~20 cells. *Indicates *P* < 0.05. Protein lysates were generated from differentiated macrophages and subjected to immunoblot with representative images shown in (**j**) for the indicated proteins. Immunoblots were quantified for LRRK2 normalised to β-actin (**k**), T73 phosphorylated Rab10 normalised to total Rab10 (**l**) and S1292 phosphorylated LRRK2 normalised to total LRRK2 (**m**). Graphs show mean ± SEM with dots indicating biological replicates (*n* = 4). *Indicates *P* < 0.05.

To confirm successful differentiation of wild type (WT), G2019S and LRRK2 knockout (KO) isogenic iPSCs to monocytes, suspended cells in the culture media were routinely collected and analysed by flow cytometry (Fig. 1a). The percentage of CD14-positive cells in the collected media varied between rounds of differentiation, however, the percentage was always similar for the different LRRK2 genotypes within each round (Fig. 1b). The percentage of CD14-positive cells in the collected media was on average 27% across the different collection timepoints, therefore immunomagnetic isolation was used to purify monocyte populations to >90% CD14-positive cells prior to experiments. Flow cytometry was used to assess the expression of additional myeloid markers with CD68 (Fig. 1c) and HLA-DR (Fig. 1d) unchanged between genotypes. In contrast, there was a small but significant reduction in the expression of CCR2 (Fig. 1e) and CD163 (Fig. 1f) in the LRRK2 KO monocytes, compared to the LRRK2 G2019S monocytes. The expression of CD16 was also measured to determine the percentage of classical (CD14^+^CD16^−^), intermediate (CD14^+^CD16^+^) and non-classical (CD14^low^CD16^+^) monocytes. This uncovered a heterogenous mix of monocyte populations that were not significantly different between the genotypes (Fig. 1g). For functional characterisation, the isolated monocytes were also differentiated to adherent macrophages and phagocytosis was assessed using fluorescent latex beads at 3 (Fig. 1h) and 16 h (Fig. 1I) timepoints. After 3 h, there was a significant reduction in the phagocytosis of the beads by the macrophages with the LRRK2 G2019S mutation compared to WT (Fig. 1h), but no difference was observed between these two genotypes at 16 h (Fig. 1i). In contrast, the LRRK2 KO macrophages showed no difference at the 3 h timepoint, but a significant reduction in the phagocytosis of the latex beads at 16 h compared to both the WT and LRRK2 G2019S macrophages (Fig. 1i). After 16 h, LRRK2 KO macrophages also had a significant reduction in the number of puncta per cell that were formed by the accumulation of fluorescent latex beads, in conjunction with a marked increase in the average area of the puncta per cell (Supplementary Fig. S1). Finally, immunoblotting was used to confirm that LRRK2 KO cells were deficient in LRRK2 protein (Fig. 1j–k). LRRK2 KO cells also had lower levels of Rab10 phosphorylation compared to LRRK2 G2019S cells, whilst Rab10 phosphorylation was not significantly different between LRRK2 G2109S and WT monocytes (Fig. 1l). Weak detection of the LRRK2 S1292 phosphorylation site could be observed only in LRRK2 G2019S cells indicative of increased LRRK2 kinase activity (Fig. 1m). These results show that monocytes/macrophages with different LRRK2 genotypes can be successfully differentiated from the iPSCs.

### Increased TLR-stimulated cytokines with the LRRK2 G2019S mutation

**Fig. 2:**
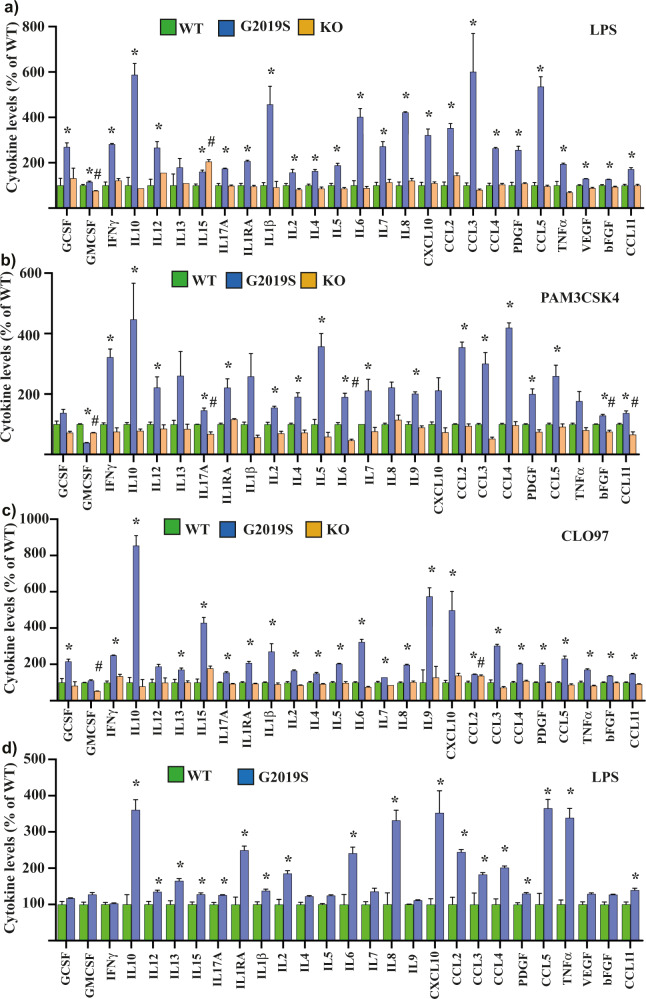
Potentiated cytokines in G2019S monocytes and macrophages. Multiplex ELISA assay was used to measure levels of the indicated inflammatory cytokines in tissue culture media from LRRK2 wild type (WT, green bars), G2019S (blue bars) and knockout (KO, orange bars) monocytes following 24 h stimulation with 500 ng/ml LPS (**a**), 1 μg/ml pam3CSK4 (**b**) and 1 μg/ml CLO97 (**c**). **d** LRRK2 wild-type and G2019S monocytes were further differentiated to macrophages and cytokines in the tissue culture media were measured following 24 h stimulation with 500 ng/ml LPS. For all graphs, the data are expressed as the percent change for a particular cytokine relative to LRRK2 wild type, which was set at 100%. Graphs show mean ± SEM and are based on *n* = 3 technical replicates and representative of at least two biological replicates. *Indicates *P* < 0.05 for G2019S compared to wild type. ^#^Indicates *P* < 0.05 for LRRK2 knockout compared to wild type.

Monocytes from the different cell lines were then stimulated with TLR agonists to determine the effect of LRRK2 genotype on the levels of cytokines released into the tissue culture media. Multivariate analysis of the measured cytokine levels in the tissue culture media 24 h after stimulation indicated a significant effect of LRRK2 genotype to influence cytokines for cells stimulated with LPS (*P* = 0.002, Fig. 2a), Pam3CSK4 (*P* = 0.001, Fig. 2b) and CLO97 (*P* = 0.019, Fig. 2c). Evaluation of the estimated marginal means clearly indicated the significant effect of genotype was driven by increased cytokine levels in the tissue culture media of cells with LRRK2 G2109S mutation. Indeed, the vast majority of cytokines were increased in the tissue culture media of LRRK2 G2019S cells for all three of the MyD88-dependent TLR agonists used (Fig. 2a–c). In contrast to LRRK2 G2019S, LRRK2 KO monocytes produced similar levels of cytokines to WT cells, with some incidences of small but significantly reduced cytokines being measured, particularly in the levels of GMCSF (Fig. 2a–c). Interestingly, when the TRIF pathway agonist Poly(I:C) was used, there was no overall significant effect of genotype on the levels of cytokines in the tissue culture media (*P* = 0.066, Supplementary Fig. S2). Increased cytokine levels were also detected in the tissue culture media of LRRK2 G2019S monocytes at an earlier (6 h) timepoint following LPS stimulation (*P* = 0.002, Supplementary Fig. S3). Intriguingly, at this earlier timepoint there was also a significant increase in GMCSF, IL1RA, IL8, CCL3, CCL4 and TNFα in the LRRK2 KO cells (Supplementary Fig. S3). There was no difference in the baseline expression of TLR4 between the different monocyte genotypes, and LPS increased TLR4 levels similarly in all genotypes (Supplementary Fig. S4A). In contrast, LPS treatment did not affect levels of LRRK2 in the differentiated WT or G2019S monocytes (Supplementary Fig. S4B). Importantly, a significant effect of genotype (*P* = 0.002) could be confirmed with the second set of available subclones for the three different cell lines stimulated with LPS, and again the effect of genotype was driven by increased cytokine levels in the tissue culture media of the cells with the LRRK2 G2019S mutation (Supplementary Fig. S5). Stimulation with LPS increased the proportion of classical monocytes (Supplementary Fig. S6A), with a reduction in intermediate (Supplementary Fig. S6B) and non-classical (Supplementary Fig. S6C) monocytes and the effect was similar across the different LRRK2 genotypes. The same result of increased cytokines/chemokines in the tissue culture for the LRRK2 G2019S compared to WT cells was also observed with 50 ng/ml LPS (multivariate *P* = 0.006, Supplementary Fig. S7), and following additional differentiation to adherent macrophages and subsequent stimulation with 500 ng/ml LPS (multivariate *P* = 0.003, Fig. 2d). The levels of cytokines in the tissue culture media from the unstimulated cells were generally below the detection level of the multiplex ELISA assay, thus, whether LRRK2 G2019S potentiates cytokine release even in the absence of TLR activation could not be determined.

### LRRK2 kinase inhibitors do not suppress TLR-stimulated cytokines

**Fig. 3:**
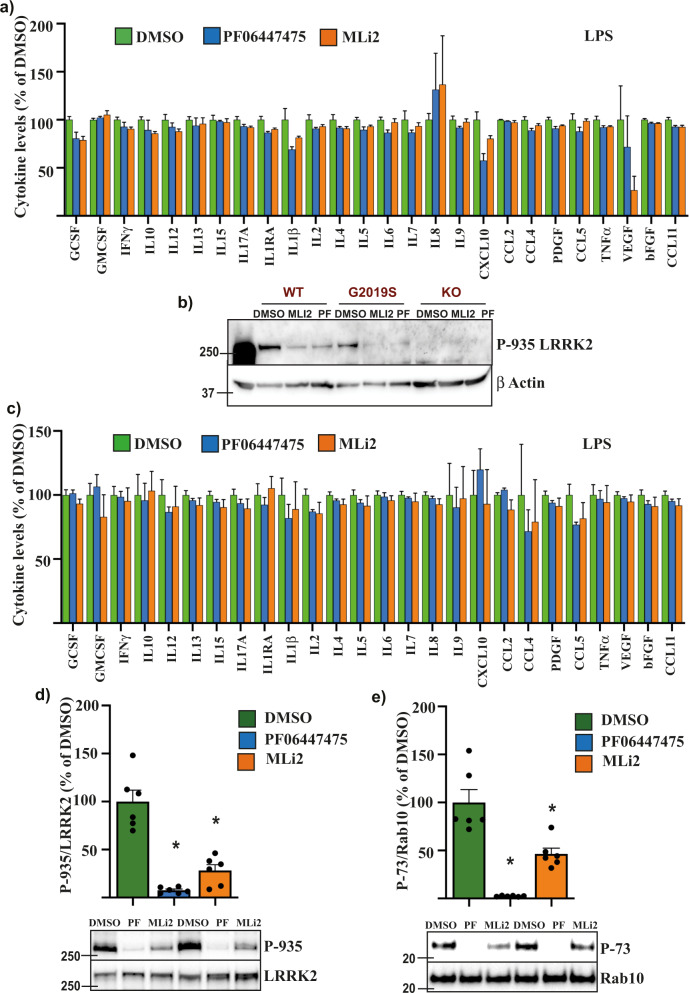
LRRK2 kinase inhibitors do not affect TLR-mediated inflammation. **a** Multiplex ELISA assay was used to measure levels of the indicated inflammatory cytokines in tissue culture media from LRRK2 G2019S cells that were treated with DMSO (green bars), or the LRRK2 kinase inhibitors PF06447475 (0.5 μM, blue bars) or MLi2 (0.1 μM, orange bars) for 24 h following stimulation with 500 ng/ml LPS. **b** Immunoblotting of the LRRK2 pharmacodynamic biomarker site, serine 935 was performed in parallel sets of differentiated monocytes. **c** Multiplex ELISA assay was used to measure levels of the indicated inflammatory cytokines in tissue culture media from primary monocytes isolated from healthy donor peripheral blood mononuclear cells were cells that were treated with DMSO (green bars), or the LRRK2 kinase inhibitors PF06447475 (0.5 μM, blue bars) or MLi2 (0.1 μM, orange bars) for 24 h following stimulation with 500 ng/ml LPS. Immunoblotting was used to measure LRRK2 serine 935 (**d**) and Rab10 threonine 73 (**e**) phosphorylation in primary monocytes isolated from healthy blood donors and treated as above (*n* = 6). Representative immunoblots are shown. Graphs show mean ± SEM and data is expressed as the percent change relative to DMSO treated cells which are set at 100%. Cytokine graphs are based on *n* = 3 technical replicates and representative of at least two biological replicates. *P < 0.05 compared to DMSO.

As the LRRK2 G2019S mutation increases the kinase activity of LRRK2, small-molecule inhibitors of LRRK2 were employed to determine their efficacy in preventing TLR-mediated cytokine release. Monocytes with the LRRK2 G2019S mutation were treated with TLR agonists in the presence or absence of MLi2 or PF06447475 for 24 h. Surprisingly, neither inhibitor had an effect on the cytokine levels in LPS-stimulated monocytes, (*P* = 0.402, Fig. 3a) or in G2019S monocytes treated with Pam3CSK4 (*P* = 0.112) (Supplementary Fig. S8A). There was also no effect of MLi2 on the tissue culture media levels of cytokines for the WT (*P* = 0.358, Supplementary Fig. S8B), or LRRK2 KO (*P* = 0.794, Supplementary Fig. S8C) cells treated with LPS. Despite a lack of effect of MLi2 on secreted cytokine levels, two-way ANOVA indicated an overall significant effect of MLi2 treatment on TLR4 expression, with an increase observed in the WT and G2019S monocytes (*P* = 0.004). However, differences in TLR4 levels between these groups were not significantly different following post hoc testing (Supplementary Fig. S9). Parallel immunoblotting for phosphorylation of the LRRK2 pharmacodynamic biomarker site serine 935 showed a marked reduction with LRRK2 inhibitors, confirming they were functional in the monocyte cell lines at the concentrations employed (Fig. 3b). In case the iPSC-derived monocytes may not fully capture all the features of primary human monocytes, the effect of the LRRK2 inhibitors was also tested in monocytes isolated from peripheral blood mononuclear cells obtained from six healthy blood donors. Isolated monocytes were treated for 24 h with LPS in the presence or absence of the LRRK2 inhibitors PF06447475 and MLi2. Multivariate analysis of the resulting data revealed a significant overall effect of inhibitor treatment on cytokine levels (*P* = 0.023), however, no significant differences were observed with post hoc testing for any of the individual cytokines (Fig. 3c). A robust reduction in both LRRK2 S935 and Rab10 T73 phosphorylation was observed in parallel immunoblots, confirming the inhibitors were functional (Fig. 3d). The LRRK2 inhibitor experiments were then repeated following differentiation of the LRRK2 G2019S monocytes into macrophages, with cells again stimulated with LPS in the presence or absence of MLi2. Consistent with the results for monocytes, MLi2 inhibitor treatment did not affect the levels of cytokines in the tissue culture media from LRRK2 G2019S differentiated macrophages stimulated with LPS (*P* = 0.350, Supplementary Fig. S10). We also treated primary monocyte-derived macrophages with TLR agonists LPS, Pam3CSK4 or R848 in the presence or absence of MLi2, PF06447475 and GSK2578215A LRRK2 inhibitors. Again, however, no significant effect of LRRK2 inhibitors was observed for any of the treatment conditions (LPS *P* = 0.937, Pam3CSK4 *P* = 0.290, R848 *P* = 0.154) despite a robust reduction in LRRK2 S935 and Rab10 T73 phosphorylation with inhibitor treatment (Supplementary Fig. S11). For all the above experiments, the LRRK2 inhibitors were employed for 24 h. Thus, we also aimed to determine the effect of more chronic LRRK2 inhibition on the levels of TLR-stimulated cytokines. Primary human monocytes were again differentiated to macrophages, but this time LRRK2 kinase inhibitors were added every 48 h throughout the 7 days of differentiation and prior to stimulation with LPS. Despite prolonged inhibitor treatment, no significant effect of LRRK2 inhibition on the levels of LPS-stimulated cytokines was observed (Supplementary Fig. S12A). In parallel immunoblots, chronic LRRK2 inhibitor treatment was associated with a significant reduction in both S935 phosphorylated LRRK2 and T73 phosphorylated Rab10 (Supplementary Fig. S12B, C). Chronic LRRK2 inhibitor treatment also had no significant effect on the total levels of LRRK2 (Supplementary Fig. S12D) or Rab10 (Supplementary Fig. S12E).

### IFNg increases LRRK2 and Rab10 T73 phosphorylation in human macrophages

**Fig. 4:**
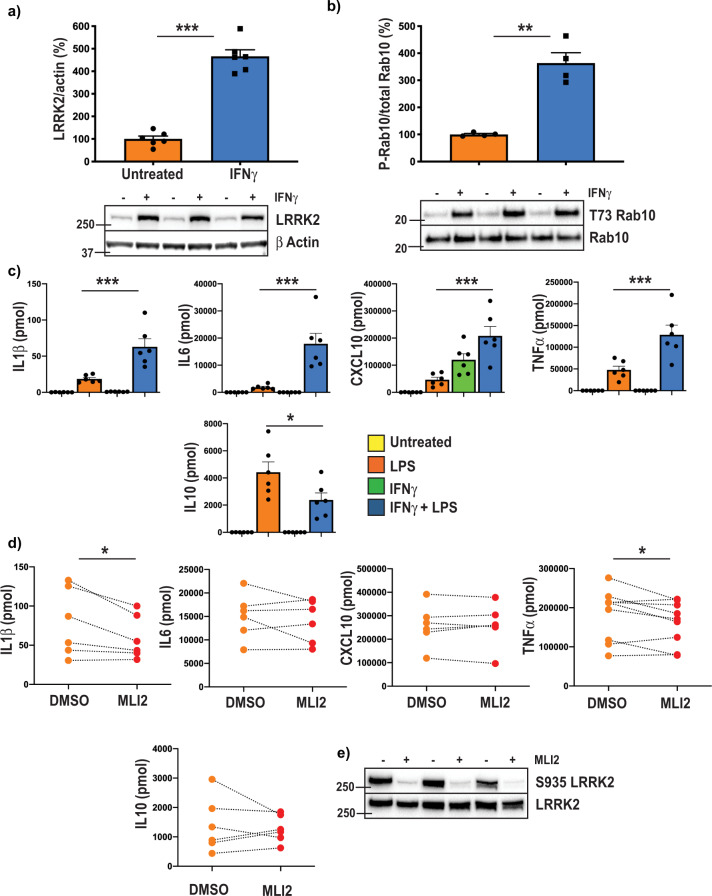
Interferon gamma increases LRRK2 levels and Rab10 phosphorylation. Primary monocytes from healthy donors were differentiated to macrophages and then treated with 100 U/ml interferon gamma for 48 h. Immunoblot analysis was then performed for LRRK2 (**a**) and total and T73 phosphorylated Rab10 (**b**). Representative immunoblots are shown. Graphs show mean ± SEM (*N* = 6) and data is expressed as the percent change relative to untreated cells, which are set at 100%. **P < 0.01, ***P < 0.001 compared to untreated cells. **c** Primary monocytes were differentiated to macrophages and then treated with or without 100 U/ml interferon gamma for 24 h followed by treatment with or without 500 ng/ml LPS for a further 24 h. Multiplex ELISA assays were used to measure levels of the indicated inflammatory cytokines in tissue culture media. Graphs show mean ± SEM (*n* = 6). *P < 0.05, ***P < 0.001, compared to cells treated only with LPS. **d** Primary monocytes were differentiated to macrophages and then treated with 100 U/ml interferon gamma for 24 h followed by 500 ng/ml LPS for a further 24 h in the presence or absence of 0.1 μM MLi2. Multiplex ELISA assays were used to measure levels of the indicated inflammatory cytokines in tissue culture media. Graphs show data from the different individuals (*n* = 6). *P < 0.05 compared to DMSO. **e** Representative immunoblot of total and S935 phosphorylated LRRK2 following treatment with or without 0.1 μM MLi2.

We next aimed to determine if LRRK2 inhibitors may show more effect in macrophages with elevated levels of LRRK2 following treatment with IFNγ. Treatment of primary monocyte-derived macrophages with IFNγ resulted in a significant approximately four times increase in the levels of LRRK2 protein (Fig. 4a), and a corresponding increase in the phosphorylation of Rab10 at T73 (Fig. 4b) compared to untreated cells. Further stimulation of IFNγ treated cells with LPS resulted in a significant increase in the levels of IL1b, IL6, CCXL10 and TNFα compared to those treated with LPS or IFNγ alone (Fig. 4c). In line with the known effect of IFNγ to induce a proinflammatory monocyte phenotype, the levels of IL10 were also significantly decreased in the dual treated macrophages (Fig. 4c). Levels of other cytokines remained unchanged between the LPS or the LPS plus IFNγ groups. MLi2 was then used to determine whether the five cytokines significantly altered with the dual-LPS plus IFNγ treatment was associated with the increased LRRK2 kinase activity in these cells. A small but significant reduction in the levels of IL1β and TNFα was observed with MLi2 treatment (Fig. 4d), whereas the levels of IL6, CXCL10 and IL10 remained unchanged. Again, LRRK2 inhibitor treatment resulted in a marked reduction in LRRK2 S935 phosphorylation (Fig. 4e).

## Discussion

Neuroinflammation has long been associated with brain pathology in PD^41^. However, there is increasing recognition that peripheral inflammation may also contribute to PD pathogenesis. This recognition is being driven by discoveries showing that proteins genetically associated with PD, such as alpha-synuclein PINK1, Parkin, LRRK2 and GBA are highly expressed in peripheral immune cells and contribute to the regulation of immunity^32^. The objective of this study was to determine how levels of LRRK2, and in particular LRRK2 kinase activity, modulate the response of human monocytes and macrophages to innate immune TLR activation, the predominate pathogen response pathway in humans. Using isogenic human iPSC-derived monocytes and macrophages, higher levels of inflammatory cytokines and chemokines were measured in tissue culture media from cells harbouring the LRRK2 G21019S mutation. Increased inflammatory cytokine levels have previously been observed in transgenic mice overexpressing LRRK2 with the R1441C^42^ and R1441G^43^ mutations and treated with the TLR4 agonist LPS. With regard to the G2019S mutation, increased cytokine levels were observed in transgenic LRRK2 G2019S mouse macrophages infected with *S. typhimurium*^23^, but increased inflammation was not observed in *S. typhimurium* infected macrophages from LRRK2 G2019S knock-in mice^29^. Potentiated peripheral cytokine levels were also not observed in mice overexpressing the G2019S mutation and treated with LPS^44^. Thus, the model and pathogenic context employed likely contribute to divergent immune response outcomes. Importantly, however, clinical studies have provided evidence for increased inflammation in asymptomatic carriers of the LRRK2 G2019S mutation^38^, and regular use of non-steroidal anti-inflammatory medication may reduce the penetrance of LRRK2-associated PD^40^.

Mechanistically, our hypothesis was that potentiated inflammation associated with the G2019S mutation would be due to the two- to threefold increase in the kinase activity of LRRK2 that the G2019S mutation imparts^45,46^. However, under the experimental conditions employed, we did not observe a robust anti-inflammatory effect of LRRK2 kinase inhibitors clearly suggesting that potentiated cytokine levels with the G2019S mutation occurred independently of LRRK2 kinase activity. These results differ from studies focused on neuroinflammation that have used microglia to demonstrate an anti-inflammatory effect of LRRK2 kinase inhibitors^47–49^. Interestingly, the expression of LRRK2 appears substantially lower in microglia compared to monocytes/macrophages^42^ and it will be of interest to determine the extent that any LRRK2 inflammatory regulating pathway may be conserved in these related but different cell types. This may be important in regard to the treatment of PD and/or understanding the mechanism of action of LRRK2 kinase inhibitors, particularly as recent rodent^29,42,50^ and clinical^51–53^ studies support an important role for peripheral myeloid cells in PD pathogenesis. In contrast, the ability to attenuate neuroinflammation without an effect on peripheral TLR-mediated immunity may even be of benefit for the clinical translation of LRRK2 inhibitors by reducing the potential for unwanted side effects.

In addition to the lack of effect of LRRK2 inhibitors, we also found no effect of LRRK2 KO on TLR-stimulated cytokines. This result is consistent with early work showing no effect on TLR-stimulated cytokine production in bone marrow-derived macrophages from LRRK2 knockout mice^18,54^. We did however observe an impairment in the phagocytosis of latex beads in the LRRK2 knockout macrophages at a longer 16 h timepoint. A recent study using a similar isogenic human iPSC-derived macrophage approach, that more comprehensively evaluated phagocytosis in response to different pathogenic stimuli did not find impaired phagocytosis with LRRK2 KO at more acute 2-h timepoints^55^. However, LRRK2 was localised to phagosomes at later stages of maturation following the induction of phagocytosis. In contrast, microglia from LRRK2 KO mice did show impaired phagocytosis via dysregulation of WAVE2 at acute timepoints^56^. Murine studies have also demonstrated that a loss of LRRK2 results in impaired clearance of *S. typhimurium* leading to increased susceptibility^23^. Worse pathological outcomes have also been measured in LRRK2 knockout mice or mouse cells infected with reovirus^29^ or *L. monocytogenes*^57^. Indeed, impaired pathogen clearance could result in prolonged low-grade inflammation with increased cytokines, such as that observed in PD patients, but this was not detected in our LRRK2 knockout macrophages due to the more acute conditions employed. Intriguingly, LRRK2 KO has conversely been associated with improved clearance of *M. tuberculosis*^27^ and ameliorated inflammation following injection of the HIV Tat protein^58^. Thus, although our findings support a role for LRRK2 to regulate pathogen clearance, the exact outcomes clearly depend on the pathogen, cell type and model used for study.

Caveats to our study should also be noted. Firstly, isogenic iPSC cell lines are a powerful tool for understanding the effects of point mutations in a human context, but they do not provide information on the biological variation in responses across a population. It will therefore be important to replicate outcomes based on a single iPSC line with isogenic correction, in other human cell lines with the LRRK2 G2019S mutation. Secondly, our study employs homogenous in vitro models that are a powerful tool for studying immune signalling in human cells, but cannot capture the crosstalk or synergy of a physiological heterogenous immune system. Indeed, different pathogenic stimuli, different LRRK2 mutation models, different inhibitor concentrations or treatment timepoints could all lead to different immune response outcomes, and thus comparison between models or a broad interpretation of results from a single model should entail caution. Further work is also required to provide mechanistic insight into how LRRK2 mutations may potentiate inflammation in the absence of a role for kinase activity. Stimulation of macrophage cells with LPS is known to induce the phosphorylation of LRRK2 at Ser935, a residue known to regulate the subcellular localisation of LRRK2^22,59,60^. It would thus be of interest to determine if/how the LRRK2 G2019S mutation alters LRRK2 localisation downstream of TLR activation. The LRRK2 G2019S mutation is also known to interfere with mitochondrial and lysosomal function^61,62^, as well as intracellular trafficking^63^, which could all in turn impact the production or release of inflammatory cytokines. Indeed, it would be important to determine if altered trafficking pathways modulate the release of cytokines into tissue culture media across the different genotypes. In summary, our data add to the emerging concept that peripheral innate immune dysfunction may contribute to PD, however further work is required to determine the mechanisms by which this occurs, and to understand the implications for LRRK2 kinase inhibitor therapies to treat PD.

## Methods

### Induced pluripotent stem cells (iPSCs)

IPSCs were derived with informed written consent from a PD patient with the G2019S mutation. Characterisation of these cells, along with the corresponding Zinc-finger gene-corrected isogenic control cell line, and LRRK2 knockout cell line on the same isogenic background have been described previously^64,65^. Two subclones of each cell line were available. Ethics approval for use of the iPSCs was provided by the University of Sydney Human Research Ethics Committee (#2017/094). IPSCs were maintained on Geltrex and cultured using Essential 8 media (both Thermofisher) with manual passaging under a stereomicroscope (Nikon SMZ1270).

### Differentiation of iPSCs to monocytes

IPSCs were differentiated into monocytes following the protocol of Yanagimachi et al.^66^, following the schematic outlined in Supplementary Fig. S13. All growth factor and cytokine supplements were purchased from Miltenyi Biotech. Cell culture supernatant containing CD14 expressing cells was collected on days 18, 22, 26 and 30 and either used immediately for experiments or cryopreserved (using RPMI supplemented with 20% FBS and 10% DMSO) for experiments later. Cryopreserved cells were allowed to recover for 2 h prior to isolation for experiments.

### Isolation of primary peripheral blood mononuclear cells (PBMCs)

For the isolation of primary PBMCs, residual buffy coat samples were obtained from healthy donors to the Australian Red Cross Blood Service. Ethics approval for studies using human blood samples was provided by the University of Sydney Human Research Ethics Committee (#2017/857). PBMCs were isolated using Ficoll-Paque (GE Healthcare) and centrifugation (400 × *g* for 30 min with acceleration 4 and brake 0) in a swing bucket rotor. Isolated PBMCs were resuspended in media and used for the immunomagnetic isolation of monocytes.

### Immunomagnetic isolation of monocytes

Monocytes were positively selected from differentiated iPSC tissue culture media or primary PBMCs using CD14 microbeads in conjunction with magnetic-activated cell sorting (MACS) as per the manufacturer instructions (Miltenyi Biotech). Monocytes were used for experiments or differentiated to macrophages over a further 7 days using RPMI-1640 (Thermofisher) supplemented with 10% FBS (Thermofisher) and 100 ng/ml MCSF.

### Cell treatments

Depending on the application, from 2.5 × 10^4^ to 1 × 10^6^ CD14^+^ cells were used for experiments. TLR agonists (Invivogen) were used at the following concentrations; lipopolysaccharide (LPS) (TLR4 agonist, 0.5 mg/ml), PAM3CSK4 (TLR1/2 agonist, 1 mg/ml), CLO97 (TLR7/8 agonist, 1 mg/ml) R848 (TLR7/8 agonist, 1 mg/ml) and Poly(I:C) (TLR3 agonist, 10 mg/ml). LRRK2 kinase inhibitors (Tocris) were used at the following concentrations; 100 nM MLi2, 500 nM PF06447475, and 500 nM GSK2578215A. Human recombinant IFNg (Stemcell Technologies) was used at 100 U/ml for 48 h.

### Multiplex cytokine ELISA assays

Cytokines were assayed using magnetic Bio-Plex Pro Human Cytokine 27-plex kits (Bio-Rad) as per the manufacturers’ instructions. ELISA assays were performed at least in triplicate and data presented as either the cytokine levels in pmol, or the percent change in cytokine levels compared to wild-type cells. Samples from different groups were distributed equally across ELISA plates. Cytokines outside the limit of detection were not included in analyses, consequently not all graphs show the complete set of 27 cytokines.

### Cell lysis and immunoblotting

Cells were lysed in buffer containing 50 mM Tris-HCL pH 7.5, 1 mM EGTA, 1 mM EDTA, 1 mM sodium orthovanadate, 50 mM sodium fluoride, 5 mM sodium pyrophosphate, 0.27 M sucrose, 1 mM benzamidine, 1 mM phenylmethylsulfonyl fluoride (PMSF) and 1% (v/v) Triton X-100 and clarified by centrifuging at 12,000 × *g* for 20 min at 4 °C. Protein concentration was determined using a bicinchoninic assay (Pierce) and samples were made up in 1× NuPAGE LDS buffer (Thermofisher). Samples were then separated using 4–12% Novex Tris-glycine gels (Thermofisher) and transferred onto nitrocellulose membrane (Biorad). Membranes were blocked with 5% skim milk powder in Tris-buffered saline with 0.1% (v/v) Tween 20 (TBST). Where indicated, membranes were probed for total LRRK2 (N241A/34, Neuromab), phosphorylated LRRK2 pSer935 (UDD2, Abcam), total Rab10 (D36C4, Cell Signaling Technology), phosphorylated Rab10 pThr73 (MJF-R21, Abcam), and β-actin as a loading control (AC-15, Abcam). Primary antibodies were used at 1:1000 dilution in 5% skim milk in TBST. Anti-mouse and anti-rabbit horseradish peroxidase secondary antibodies (Biorad) were used at 1:5000 dilution in 2.5% skim milk in TBST. Enhanced chemiluminescence reagent (GE Healthcare) and a Chemidoc MP digital imaging system were used for detection. Imagelab software (Biorad) was used for quantification with proteins of interest normalised to β-actin. Blots were derived from the same experiment and processed in parallel.

### Flow cytometry

Cells were pelleted by centrifugation (300 × *g* for 4 min at 4 °C), washed with fluorescence-activated cell sorting (FACS) buffer (1× PBS,1 mM EDTA, 25 mM HEPES, and 1% heat-inactivated FBS, pH 7.4) and then incubated with FcR Blocking Reagent (Miltenyi Biotech) for 10 min at 4 °C. Cells were washed as above and then resuspended in FACS buffer with fluorochrome-conjugated monoclonal antibodies for 20 min at 4 °C. The antibodies used were PE-Cy7-conjugated anti-CD14, BV421-conjugated anti-CD16, BV510-conjugated anti-CCR2, BV711-conjugated anti-CD68, PE-CF594-conjugated anti-CD163 (all from Becton Dickinson), and PE-conjugated anti-TLR4 and Alexa Fluor 488-conjugated anti-HLA-DR (both from BioLegend). Following antibody incubation, cells were fixed with 2% PFA for 10 min, washed again with FACS buffer and finally resuspended in 350 ml FACS buffer for acquisition. Data were acquired using an LSR Fortessa flow cytometer (Becton Dickinson) and analysed using FlowJo software (TreeStar). At least 10,000 events were captured per condition.

### Phagocytosis assay

A 4% solution of GFP-labelled latex beads with a mean particle size of 0.5 mm (#L5530) was used to assess phagocytosis. Macrophages were incubated for 3 h and 16 h, washed with live-cell imaging solution (Thermofisher) and stained with CellMask Deep Red (Thermofisher) as per manufacturer instructions. After 15 min, cells were fixed with 4% paraformaldehyde and DAPI was used to stain the nucleus. Fluorescence intensity was measured using a C2 confocal microscope at ×40 magnification (Nikon). Fiji ImageJ particle analysis tool was then used to determine the average intensity, average number and average area of fluorescent puncta formed by the accumulation of latex beads per CellMask positive cell. CellMask was also used to exclude extracellular signals. At least five images were measured per cell line with at least ~20–50 cells analysed per image.

### Statistical analysis

Statistical analysis was performed using SPSS (IBM) or Prism (GraphPad). As different cytokines were expressed at markedly different levels, ELISA data were standardised by expressing cytokine levels as a percentage compared to WT or DMSO treated cells as appropriate. Multivariate analysis was then used to determine the effect of genotype or inhibitor treatment on cytokine levels. A significant overall effect was accepted at *P* < 0.05 using Wilks’ Lambda. Post hoc pairwise comparisons of the estimated marginal means were performed to determine significant differences compared to the wild type or DMSO treated cells. For some comparisons, where indicated, one-way ANOVA with Dunnett’s post hoc test, two-way ANOVA with the Tukey post hoc test, or *t* tests were used. For all analyses, significance was accepted at *P* < 0.05. Graphs show mean ± SEM.

### Reporting summary

Further information on research design is available in the Nature Research Reporting Summary linked to this article.

## Supplementary information

Supplementary Figures

Reporting Summary

## Data Availability

The datasets generated during and/or analysed during this study are available from the corresponding author on reasonable request.
