# Supplementary material for: Effect of LRRK2 protein and activity on stimulated cytokines in human monocytes and macrophages
Source: NPJ Parkinsons Dis. 2022 Mar 28;8:34. doi: 10.1038/s41531-022-00297-9 (PMC8960803; doi:10.1038/s41531-022-00297-9)
Supplement: Supplementary file 1 — Supplementary Figures [file 41531_2022_297_MOESM1_ESM.pdf]

Effect of LRRK2 protein and activity on stimulated cytokines in human monocytes and macrophages.

Diba Ahmadi Rastegar[1*], Laura P. Hughes[1*], Gayathri Perera[1], Shikara Keshiya[1], Siying Zhong[1], Jianqun Gao[1], Glenda M. Halliday[1], Birgitt Schüle[2], Nicolas Dzamko[1#]

1. University of Sydney, Brain and Mind Centre and Faculty of Medicine and Health, School of Medical Sciences, Camperdown, NSW, 2050, Australia.
2. Department of Pathology, Stanford School of Medicine, Stanford, CA, 94305, USA.

* both authors contributed equally to this work.

Correspondence to: Nicolas Dzamko nicolas.dzamko@sydney.edu.au School of Medical Sciences, University of Sydney, Camperdown, NSW, 2050, Australia

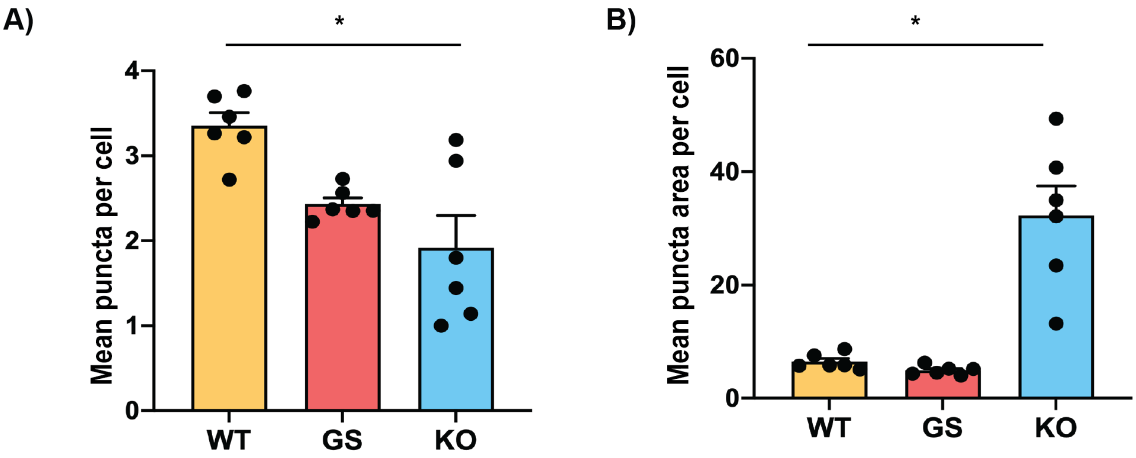

Figure S1. Impaired measures of phagocytosis in LRRK2 KO macrophages.

The uptake of GFP-labelled latex beads was used to determined the mean number of puncta per cell (**A**), and the mean area of puncta per cell (**B**), calculated using the Image J particle analysis plug in. Cell mask was used to visualize internalized GFP-signal. Graphs indicate mean ± SEM. Dots indicate the results from each image analyzed, with each image comprising at least 20 cells. * indicates $p < 0.05$.

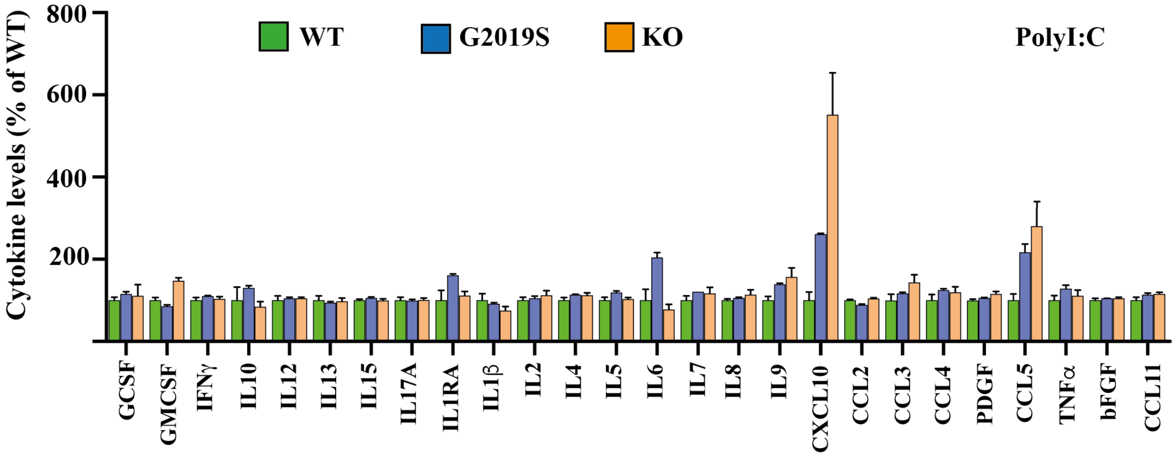

Figure S2. TLR3 stimulated cytokine levels are not affected by LRRK2 genotype. Multiplex ELISA assay was used to measure levels of the indicated inflammatory cytokines in tissue culture media from LRRK2 wild type (WT, green bars), G2019S (blue bars) and knockout (KO, orange bars) monocytes following 24 h stimulation with 10 μg/ml Poly(I:C) Data is expressed as the percent change for a particular cytokine relative to LRRK2 wild type, which was set at 100%. Graphs show mean ± SEM and are based on n = 3 technical replicates and representative of at least 2 biological replicates.

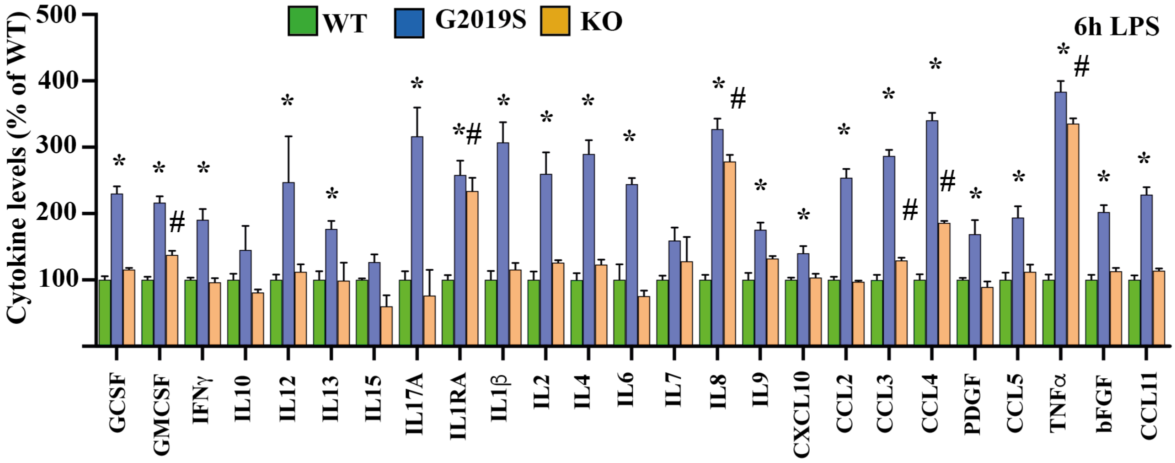

Figure S3. Increased cytokines in LRRK2 G2019S monocytes at an earlier timepoint. Multiplex ELISA assay was used to measure levels of the indicated inflammatory cytokines in tissue culture media from LRRK2 wild type (WT, green bars), G2019S (blue bars) and knockout (KO, orange bars) monocytes following 6 h stimulation with 500 ng/ml LPS. Data is expressed as the percent change for a particular cytokine relative to LRRK2 wild type, which was set at 100%. Graphs show mean ± SEM and are based on n = 3 technical replicates and representative of at least 2 biological replicates. * indicates $p < 0.05$ for G2019S compared to wild type. # indicates $p < 0.05$ for LRRK2 knockout compared to wild type.

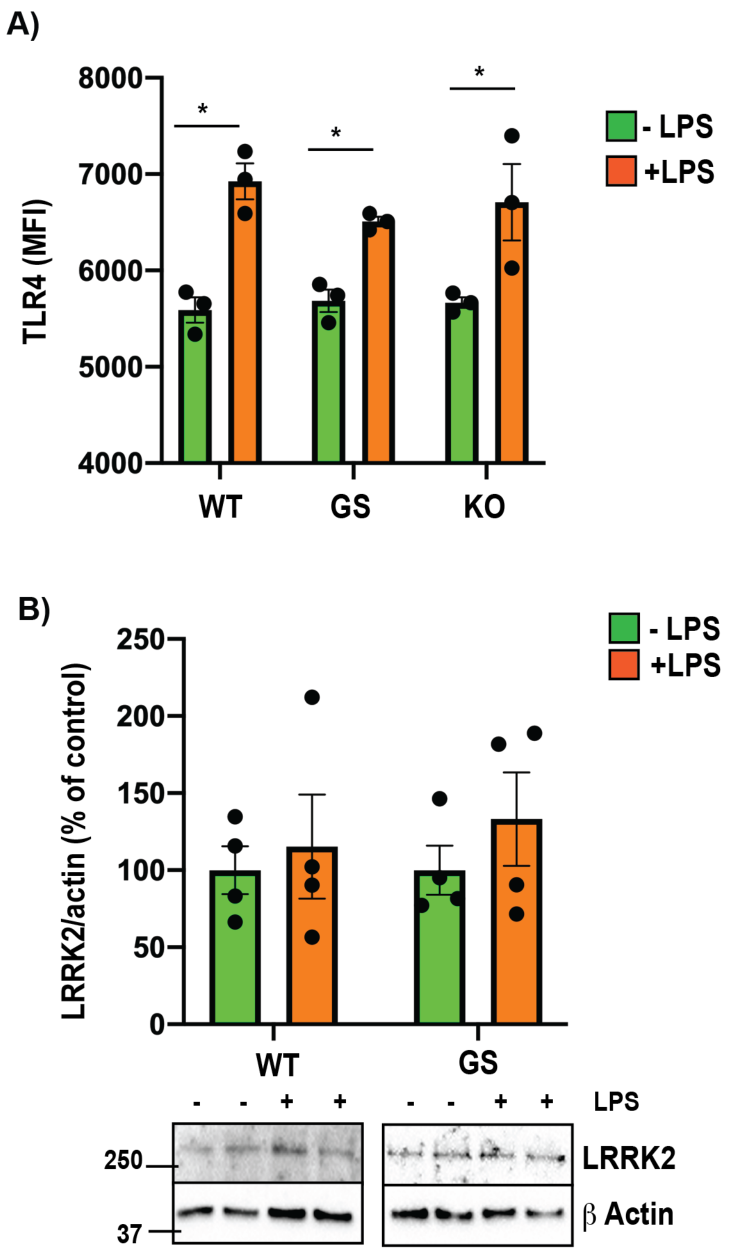

Figure S4. TLR4 and LRRK2 expression in LPS stimulated monocytes.

A) Flow cytometry was used to assess the expression of TLR4 in differentiated monocytes with the different genotypes in the presence of LPS (500 ng/ml for 24 h). Graphs show mean ± SEM and are based on n = 3 technical replicates and representative of at least 2 biological replicates. B). Immunoblotting was used to assess the expression of LRRK2 in differentiated WT and LRRK2 G2019S monocytes following stimulation with LPS (500 ng/ml for 24 h). Representative immunoblots are shown. LRRK2 expression was normalized using β-actin and expressed as a percent change relative to the unstimulated control. Graphs show mean ± SEM, n = 4.

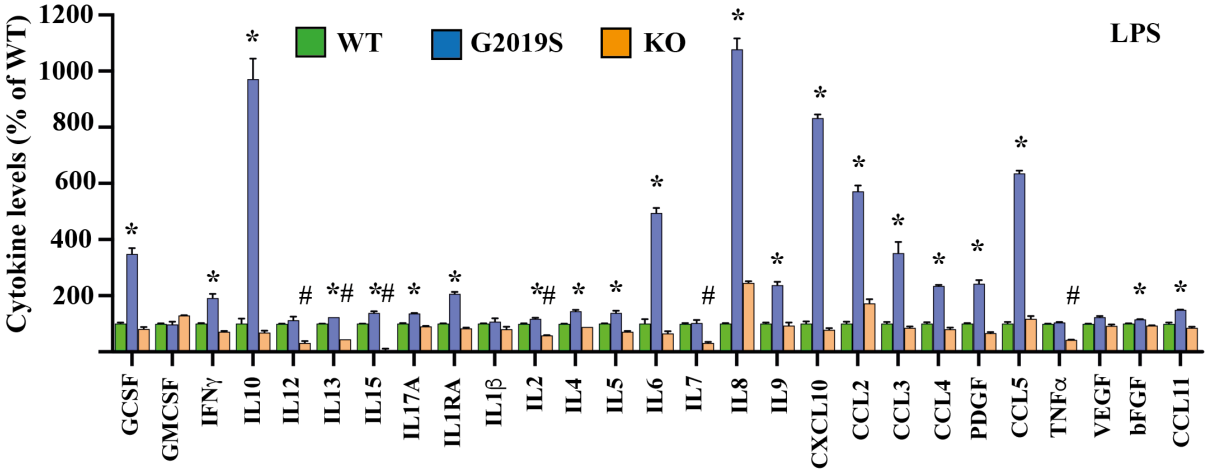

Figure S5. Increased cytokines in LRRK2 G2019S monocytes with a second clone. Multiplex ELISA assay was used to measure levels of the indicated inflammatory cytokines in tissue culture media from LRRK2 wild type (WT, green bars), G2019S (blue bars) and knockout (KO, orange bars) monocytes following 24 h stimulation with 500 ng/ml LPS. Data is expressed as the percent change for a particular cytokine relative to LRRK2 wild type, which was set at 100%. Graphs show mean ± SEM and are based on n = 3 technical replicates and representative of at least 2 biological replicates. * indicates $p < 0.05$ for G2019S compared to wild type. # indicates $p < 0.05$ for LRRK2 knockout compared to wild type.

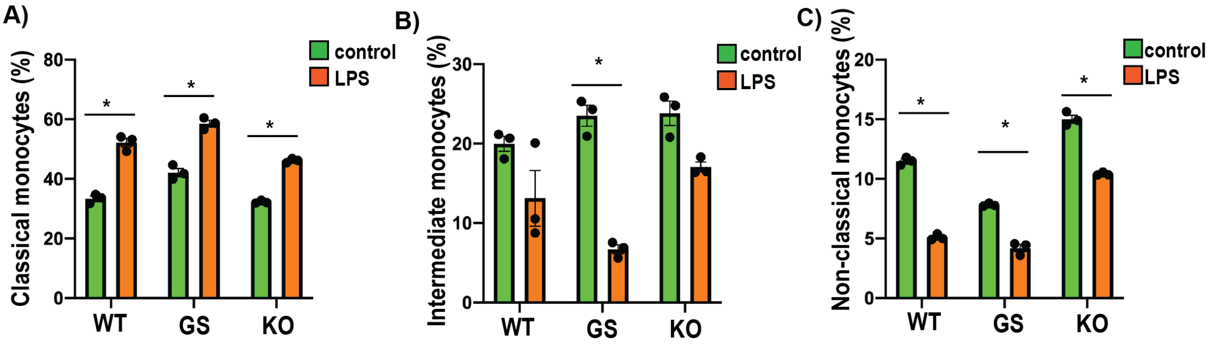

Figure S6. Effect of LPS on monocyte subpopulations.

Differentiated iPS-derived monocytes were treated with or without 500 ng/ml LPS for 24 h and the expression of CD14 and CD16 was used to determine the percentage of **(A)** classical (CD14+ CD16-), **(B)** intermediate (CD14+CD16+) and **(C)** non-classical monocytes. The effect of LRRK2 genotype or LPS treatment on the monocyte subpopulations was assessed by 2-way ANOVA. Graphs show mean ± SEM. * = $p < 0.05$. n=3.

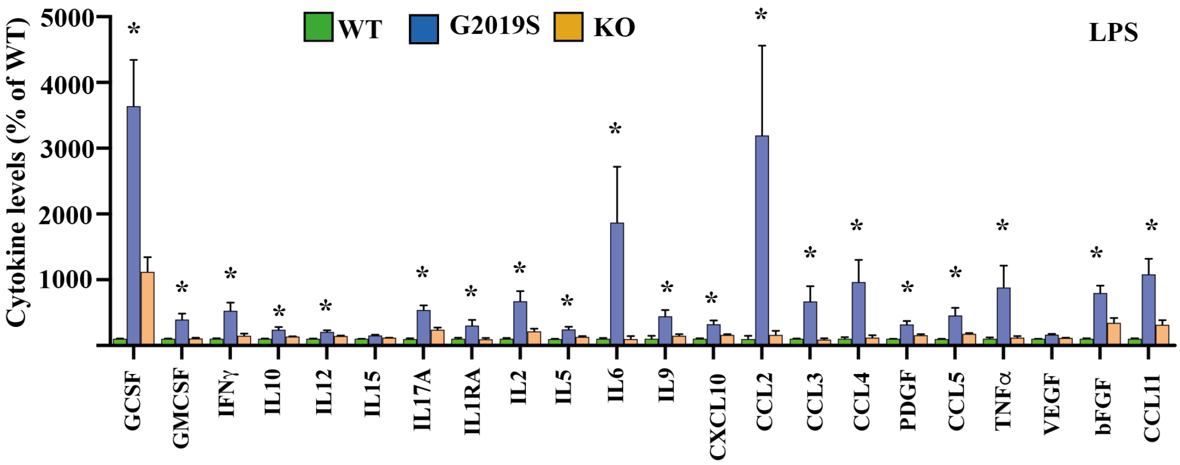

Figure S7. Increased cytokines in LRRK2 G2019S macrophages with 50 ng/ml LPS. Multiplex ELISA assay was used to measure levels of the indicated inflammatory cytokines in tissue culture media from LRRK2 wild type (WT, green bars), G2019S (blue bars) and knockout (KO, orange bars) differentiated macrophages following 24 h stimulation with 50 ng/ml LPS. Data is expressed as the percent change for a particular cytokine relative to LRRK2 wild type, which was set at 100%. Graphs show mean ± SEM and are based on n = 3 technical replicates and representative of at least 2 biological replicates. * indicates $p < 0.05$ for G2019S compared to wild type.

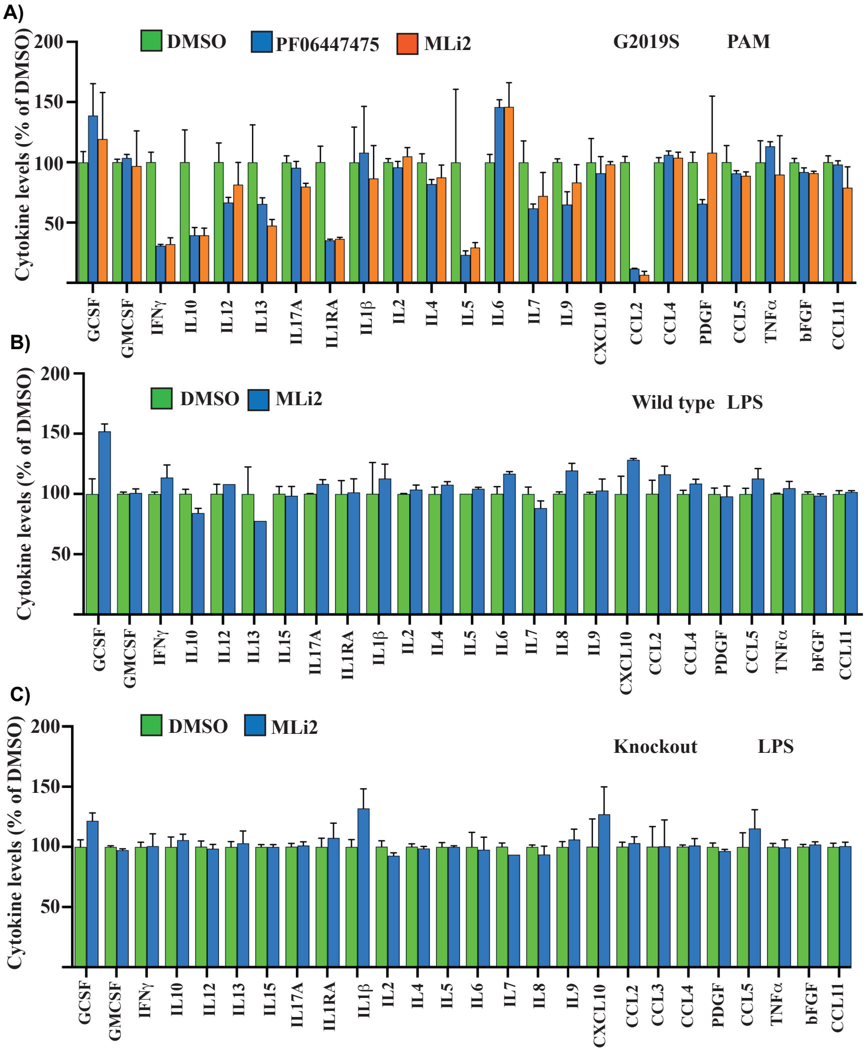

Figure S8. LRRK2 kinase inhibitors do not affect TLR-stimulated cytokines in iPSC-derived monocytes. A) Multiplex ELISA assay was used to measure levels of the indicated inflammatory cytokines in tissue culture media from LRRK2 G2019S monocytes that were treated with DMSO (green bars), or the LRRK2 kinase inhibitors PF06447475 (0.5 µM, blue bars) or MLi2 (0.1 µM, orange bars) for 24 h following stimulation with 1 µg/ml Pam3CSK4. **C**) Multiplex ELISA assay was used to measure levels of the indicated inflammatory cytokines in tissue culture media from LRRK2 WT (**B**) or LRRK2 KO (**C**) monocytes treated with DMSO (green bars), or the LRRK2 kinase inhibitor MLi2 (0.1 µM, blue bars) for 24 h following stimulation with 500 ng/ml LPS. Graphs show mean ± SEM and data is expressed as the percent change relative to DMSO treated cells which are set at 100%. Cytokine graphs are based on n = 3 technical replicates and representative of at least 2 biological replicates.

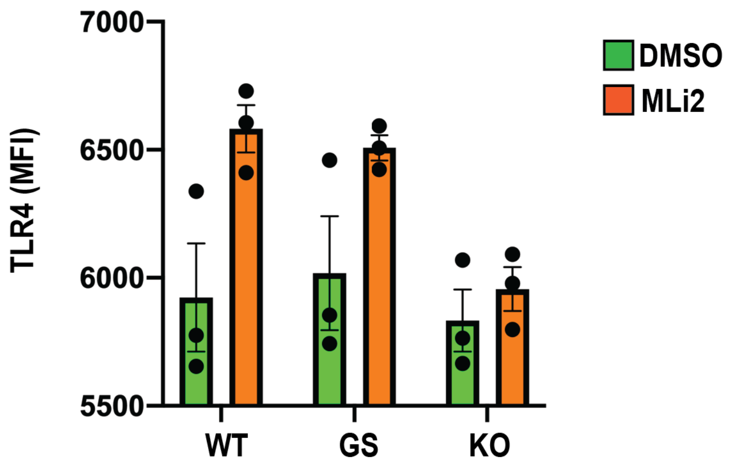

Figure S9. Effect of MLI2 on TLR4 expression.

Differentiated iPS-derived monocytes were treated with or without 0.1 μM MLi2 for 24 h and the levels of TLR4 expression measured by flow cytometry. The effect of LRRK2 genotype or LRRK2 inhibitor treatment on the levels of TLR4 was assessed by 2-way ANOVA. Graphs show mean ± SEM.

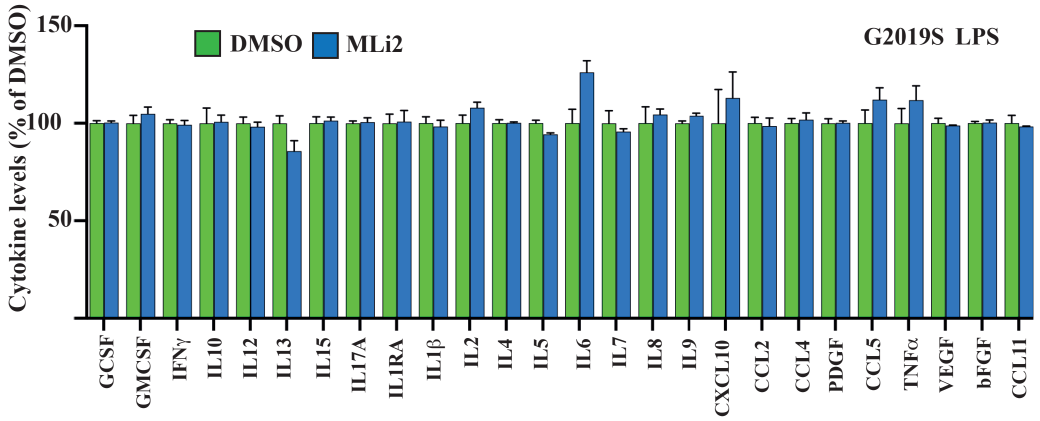

Figure S10. LRRK2 kinase inhibitors do not affect TLR4-stimulated cytokines in iPSC-derived macrophages. A) Multiplex ELISA assay was used to measure levels of the indicated inflammatory cytokines in tissue culture media from LRRK2 G2019S macrophages that were treated with DMSO (green bars), or MLi2 (0.1 µM, blue bars) for 24 h following stimulation with 500 ng/ml LPS. Graphs show mean ± SEM and data is expressed as the percent change relative to DMSO treated cells which are set at 100%. Cytokine graphs are based on n = 3 technical replicates and representative of at least 2 biological replicates.

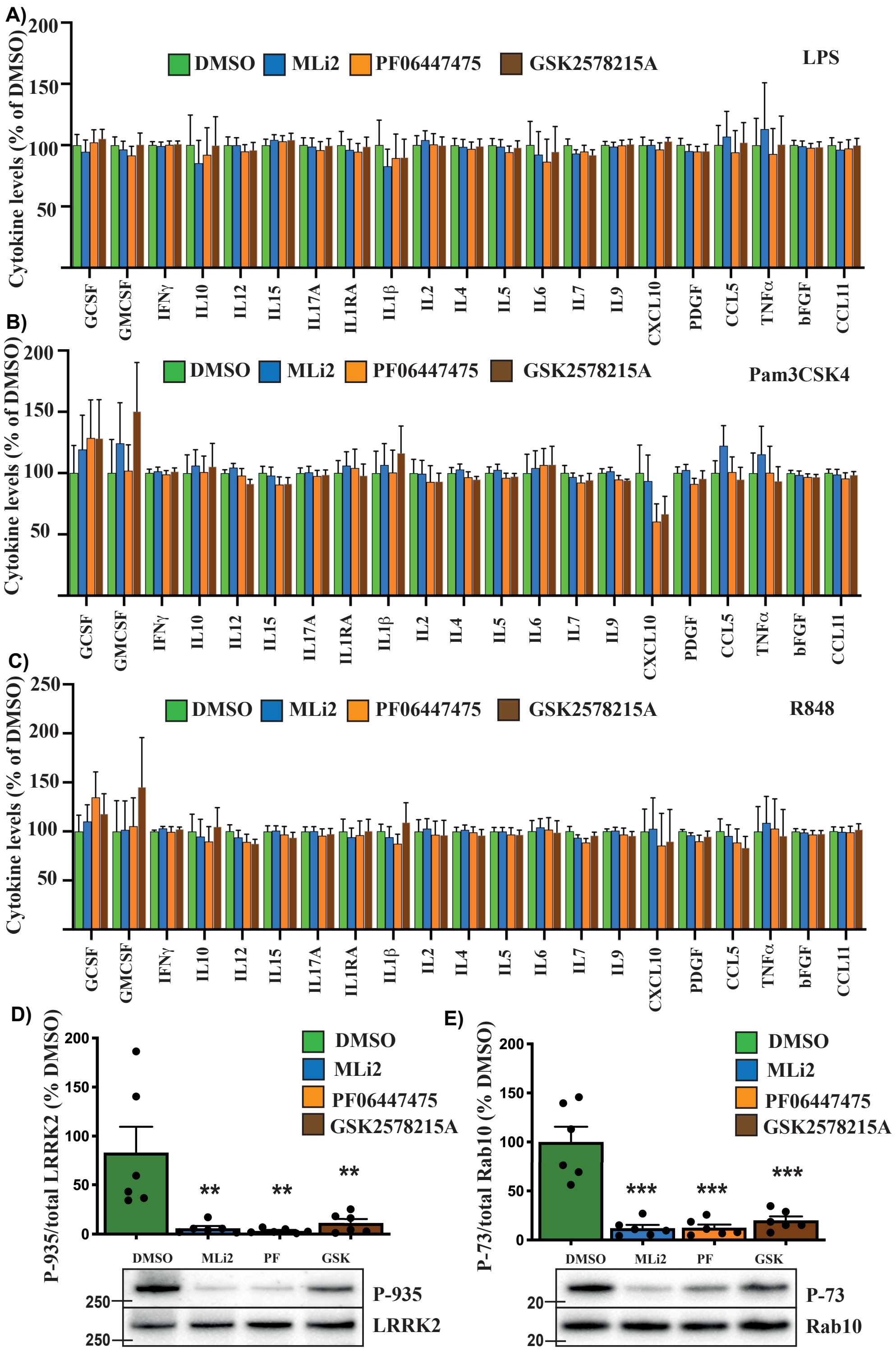

Figure S11. LRRK2 kinase inhibitors do not affect TLR4-stimulated cytokines in primary macrophages. Multiplex ELISA assay was used to measure levels of the indicated inflammatory cytokines in tissue culture media from primary monocyte derived macrophages that were treated with DMSO (green bars), or the LRRK2 kinase inhibitors MLi2 (0.1 µM, blue bars), PF06447475 (0.5 µM, orange bars) and GSK2578215A (0.5 µM, brown bars) for 24 h following stimulation with 500 ng/ml LPS (**A**), 1 µg/ml Pam3CSK4 (**B**) or 1 µg/ml R848

(**C**). Immunoblotting was used to measure LRRK2 serine 935 (**D**) and Rab10 threonine 73 (**E**) phosphorylation in primary monocytes isolated from healthy blood donors and treated as above (n=6). Representative immunoblots are shown. Graphs show mean ± SEM and data is expressed as the percent change relative to DMSO treated cells which are set at 100%. N = 6 ** = $p < 0.01$ compared to DMSO. *** = $p < 0.001$ compared to DMSO.

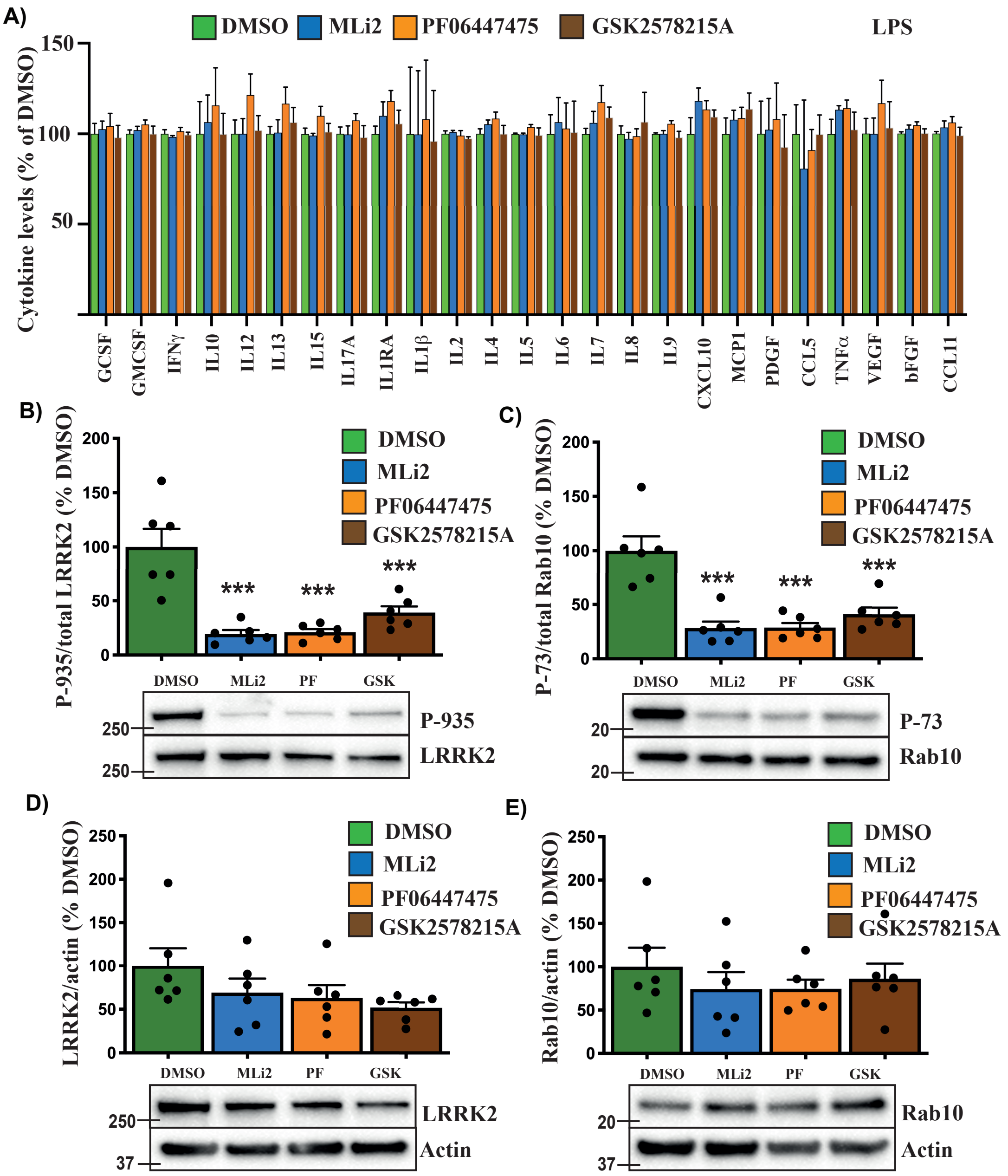

Figure S12. LRRK2 kinase inhibitors do not affect TLR4-stimulated cytokines in chronically treated primary macrophages. A) Multiplex ELISA assay was used to measure levels of the indicated inflammatory cytokines in tissue culture media from primary monocyte derived macrophages that were treated for 7 days with DMSO (green bars), or the LRRK2 kinase inhibitors MLi2 (0.1 µM, blue bars), PF06447475 (0.5 µM, orange bars) and GSK2578215A (0.5 µM, brown bars) with inhibitors replenished every 48 h. Cells were then stimulated with 500 ng/ml LPS for 24 h. Graphs show mean ± SEM and data is expressed as the percent change relative to DMSO treated cells which are set at 100%. Immunoblotting was used to measure LRRK2 serine 935 (**B**) and Rab10 threonine 73 (**C**) phosphorylation, as well

as total LRRK2 (**D**) and total Rab10 (**E**) in primary monocytes isolated from healthy blood donors and treated as above (n=6). Representative immunoblots are shown. *** = $p < 0.001$ compared to DMSO.

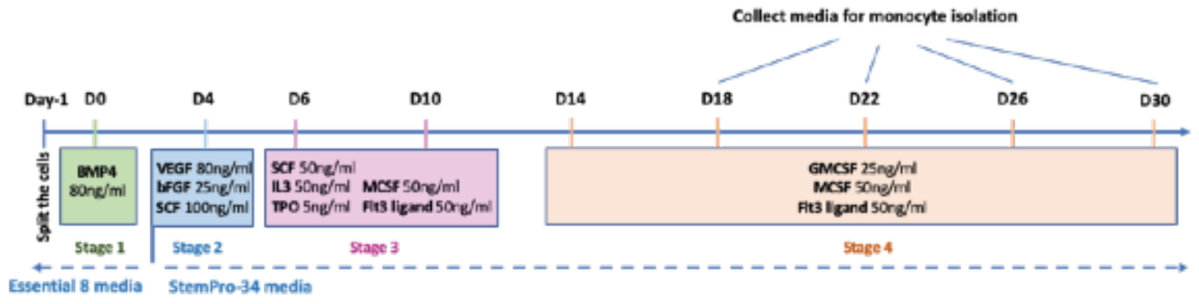

Figure S13. Differentiation of iPSC to monocytes.

The schematic outlines the differentiation of iPSC to monocytes. Briefly 80 ng/ml of bone morphogenic protein 4 (BMP4) in Essential 8 media was used to differentiate the iPSCs toward a mesodermal lineage. On day 4, the Essential 8 media was changed to StemPro-34 media supplemented with 80 ng/ml vascular endothelial growth factor (VEGF) and 100 ng/ml stem cell factor (SCF) for the development of hemoangiogenic progenitors and 25 ng/ml basic fibroblast growth factor (bFGF) to facilitate the development of mesodermal hematopoietic progenitors. On day 6 and again on day 10, StemPro-34 media was replaced with fresh media, supplemented with 50 ng/ml SCF, 50 ng/ml interleukin 3 (IL3), 5 ng/ml thyroid peroxidase (TPO), 50 ng/ml monocyte colony stimulating factor (MCSF) and 50 ng/ml Flt3 ligand to generate hematopoietic cells. On days 14, 18, 22 and 26, 50 ng/ml Flt-3 ligand, 25 ng/ml GMCSF and 50 ng/ml MCSF were supplemented into the StemPro-34 media to generate mononuclear phagocytes. Media was collected for the isolation of monocytes on the indicated days.
